# Loss of Single-Stranded DNA Binding Protein 2 Expression Is Associated with Aggressiveness and Poor Overall Survival in Patients with Invasive Breast Carcinoma

**DOI:** 10.3390/diagnostics12020487

**Published:** 2022-02-14

**Authors:** Hosub Park, Seungyun Jee, Hwangkyu Son, Hyebin Cha, Seongsik Bang, Hyunsung Kim, Su-Jin Shin, Chihwan Cha, Min Sung Chung, Jaekyung Myung, Seung Sam Paik

**Affiliations:** 1 Department of Pathology, Seoul Hospital, Hanyang University College of Medicine, Seoul 04763, Korea; parkhstm@gmail.com (H.P.); jee.seung.yun@gmail.com (S.J.); ganzi4900@gmail.com (H.S.); cnbin0111@gmail.com (H.C.); grypony@naver.com (S.B.); hhnt5841@gmail.com (H.K.); 2 Department of Pathology, Gangnam Severance Hospital, Yonsei University College of Medicine, Seoul 06273, Korea; charm@yuhs.ac; 3 Department of Surgery, Seoul Hospital, Hanyang University College of Medicine, Seoul 04763, Korea; chachihwan@gmail.com (C.C.); bovie@hanyang.ac.kr (M.S.C.)

**Keywords:** single-stranded DNA binding protein 2, invasive breast carcinoma, prognosis

## Abstract

Background: Single-stranded DNA binding protein 2 (*SSBP2*) is involved in the DNA damage response and the maintenance of genome stability. Previous studies have suggested that *SSBP2* has a tumor suppressor function or oncogenic function. Loss of *SSBP2* expression has been reported in various tumors. However, the role of *SSBP2* expression in invasive breast carcinoma has not been reported. Methods: Immunohistochemical staining for *SSBP2* was performed on tissue microarrays consisting of 491 invasive breast carcinoma cases. The result of nuclear *SSBP2* staining was stratified as either negative or positive. Then, we investigated the correlations between *SSBP2* expression and various clinicopathological parameters and patient outcomes. Results: Loss of nuclear *SSBP2* expression was observed in 61 cases (12.4%) of 491 invasive breast carcinomas. Loss of nuclear *SSBP2* expression was significantly correlated with larger tumor size (*p* < 0.001, chi-squared test), higher histological grade (*p* = 0.016, Cochran–Armitage trend test), higher pathological T stage (*p* < 0.001, Cochran–Armitage trend test), estrogen receptor status (*p* < 0.001, chi-squared test), and molecular subtype (*p* < 0.001, chi-squared test). Kaplan–Meier survival analysis revealed that patients with loss of nuclear *SSBP2* expression had worse overall survival (*p* = 0.013, log-rank test). However, loss of nuclear *SSBP2* expression was not correlated with recurrence-free survival (*p* = 0.175, log-rank test). Conclusions: Loss of nuclear *SSBP2* expression was associated with adverse clinicopathological characteristics and poor patient outcomes. *SSBP2* acts as a tumor suppressor in invasive breast carcinoma and may be used as a prognostic biomarker.

## 1. Introduction

Breast cancer is the most common malignant neoplasm and the leading cause of cancer mortality among women in Western countries as well as the leading contributor to the global cancer incidence rate in 2020 [1]. In 2020, estimated new breast cancer cases totaled 2.3 million, or 11.7% of all new cancer cases, and breast cancer was the fifth leading cause of cancer mortality worldwide, causing 685,000 deaths [1]. Breast cancer is a genetically and clinically heterogeneous disease. Although there have been marked advances in understanding breast cancer development and cancer biology, the specific treatment problem persists [2]. Various clinicopathological parameters, such as histological grade, estrogen receptor (ER), progesterone receptor (PR), human epidermal growth factor receptor 2 (HER2) gene amplification, and American Joint Committee on Cancer (AJCC) stage are currently considered in the prognosis and management of breast cancer [3]. Using proven clinicopathological prognostic factors, various proteins have been proposed as potential prognostic biomarkers of breast cancer [4].

Molecular subtype has an important influence on patient management. Molecular subtypes can be classified using surrogate markers of ER, PR, HER2, and Ki-67 immunostaining. The luminal subtype with positive ER and PR has a better prognosis than the HER2 or triple negative subtypes. Luminal subtype is divided into luminal A subtype, which has a good prognosis, and luminal B subtype, which has relatively a poor prognosis. Luminal B subtype shows a relatively high Ki-67 index compared to luminal A subtype. Some groups of luminal B subtype show HER2 positivity. The HER2 subtype is ER-negative and has HER2 overexpression. This subtype can be treated by target therapy for HER2. Triple negative subtype is negative for ER, PR, and HER2, and this subtype has a worst prognosis [5].

The single-stranded DNA binding protein 2 (*SSBP2*) gene, which is located at chromosome 5q14.1, was previously identified as a candidate tumor suppressor in myeloid leukemia patients [6]. The *SSBP2* gene is a subunit of the ssDNA-binding complex and is involved in the maintenance of hematopoietic stem cells and the maintenance of genome stability [6,7]. *SSBP2* binds to the transcriptional adaptor protein, Lim domain binding protein 1 (LDB1), through a highly conserved N-terminal domain and enhances LDB1 stability to regulate gene expression [8]. LDB1 is a protein that is reported to be involved in tumorigenesis in leukemia, head and neck cancer, and colon cancer [9]. The role of *SSBP2* in human malignancies has been studied in several solid tumors and myeloid leukemia [10,11,12,13,14,15,16]. Regarding whether *SSBP2* function is a tumor suppressor or tumor promoter, its exact role remains unclear. Several studies have demonstrated that *SSBP2* is a tumor suppressor in solid tumors and myeloid leukemia [10,11,14,15,16]. However, a few studies have suggested that *SSBP2* is a tumor promoter in glioblastoma and hepatocellular carcinoma [12,13]. The role of *SSBP2* in human breast cancer has not yet been reported.

In this study, we investigated the expression of *SSBP2* by immunohistochemistry in invasive breast carcinoma tissues, analyzed the associations between *SSBP2* expression and various clinicopathological characteristics, and assessed whether *SSBP2* is a prognostic factor for patient survival.

## 2. Materials and Methods

### 2.1. Patients and Tumor Samples

We enrolled a consecutive series of 541 patients with invasive breast carcinoma. All cases were diagnosed and underwent surgery at Hanyang University Hospital (Seoul, South Korea) between February 2003 and January 2017. We excluded patients with incomplete clinical follow-up data or no available paraffin blocks and started the study with 491 cases of invasive breast carcinoma, consisting of 471 invasive breast carcinomas of no special type, 19 invasive lobular carcinomas, and 1 case of adenoid cystic carcinoma. The baseline characteristics of included patients are summarized in Table 1. The mean age of patients was 52.9 years, and the mean follow-up period was 77 months. Of the included 491 cases, 104 cases were histological grade 1, 225 cases were histological grade 2, and 162 cases were histological grade 3. According to the eighth edition of the AJCC system, 183 cases were stage I, 207 cases were stage II, 92 cases were stage III, and 9 cases were stage IV. All tissue samples were fixed in formalin and embedded in paraffin. We reviewed all slides stained with hematoxylin and eosin (H&E) together with pathology reports and other medical records. The assessed clinicopathological characteristics were patient age, tumor size, histological grade, pathological T (pT) stage, pathological N (pN) stage, AJCC stage, lymph node metastasis, distant metastasis, ER and PR status, HER2 status, molecular subtype, and patient survival. This study was approved by the Institutional Review Board of the Hanyang University Hospital (HYUH 2021-12-014-001), and the requirement to collect informed consent was waived.

### 2.2. Tissue Microarray (TMA) Construction

A manual tissue microarrayer (Unitma, Seoul, South Korea) was used for TMA construction from archival formalin-fixed and paraffin-embedded tissue blocks. The most representative non-necrotic central portion of the tumor was selected by light microscopy. We punched a tissue cylinder 3 mm in diameter from a previously marked lesion of each donor block and transferred it to the recipient block (Unitma, Seoul, South Korea). Each TMA block comprised 6 × 5 samples.

### 2.3. Immunohistochemical (IHC) Staining

We performed the IHC staining for *SSBP2* on 4 μm thick sections from the TMA blocks. All TMA sections were deparaffinized in xylene. The deparaffinized sections were then rehydrated by a series of 5 min washes in 100%, 90%, and 75% ethanol and phosphate-buffered saline (PBS). To retrieve the antigen, the sections were heated in sodium citrate buffer (pH, 6.0) in an autoclave at 100 °C for 20 min. Then, we blocked endogenous peroxidase activity with peroxidase blocking solution (S2023; Dako, Glostrup, Denmark). The TMA slides were incubated with a rabbit monoclonal *SSBP2* antibody (1:100 dilution, ab177944; Abcam, Cambridge, UK) at 4 °C overnight, then incubated with a labeled polymer (EnVision/HRP, K5007; Dako, Glostrup, Denmark) for 30 min at room temperature. Monoclonal mouse anti-ER (Novocastra Laboratories, Newcastle, UK), monoclonal mouse anti-PR (Novocastra Laboratories, Newcastle, UK), monoclonal mouse anti-c-erbB-2 (Novocastra Laboratories, Newcastle, UK), and monoclonal mouse Ki-67 antibody (Novocastra Laboratories, Newcastle, UK) were diluted 1:50, 1:100, 1:800, and 1:100 in goat serum, respectively. Next, 3,3′-diaminobenzidine tetrahydrochloride was used as a chromogen for visualization, and counterstained with Mayer’s hematoxylin.

### 2.4. Interpretation of IHC Staining and Molecular Subtypes

*SSBP2* expression was evaluated according to the nuclear staining extent of tumor cells using a light microscope by two pathologists (H.P. and S.P.) who were blinded to the clinicopathological parameters and the patient clinical outcomes. According to previous reports [14,16], we subdivided the patients into a positive subgroup (proportion of positive tumor cells >10% of the total tumor cells) and a negative subgroup (proportion of positive tumor cells <10% of the total tumor cells).

ER and PR status was interpreted by Allred score for nuclear staining, according to ASCO/CAP guidelines. Intensity scores of 0, 1, 2, and 3 were given. Score 0 was completely negative and score 3 was strong. A proportion score of 0 to 5 was given to 0%, <1%, 1–10%, 11–33%, 34–66%, and 67–100%, respectively. When the sum of the intensity score and proportion score was 0 and 2, it was interpreted as negative, and when the sum of those scores was 3 or more, it was interpreted as positive.

HER2 status was interpreted according to the ASCO/CAP guidelines. The case where strong membranous stain was observed in more than 10% of cells in immunohistochemical staining was interpreted as positive. When weak to moderate membranous stain was observed, dual probe SISH was performed. In the SISH, HER2/CEP17 ratio of 2 or more and HER2 signal of 4 or more per cell were interpreted as positive.

Ki-67 was read by eyeballing the percentage of cells showing nuclear stain from 0% to 100% in 10% increments.

Molecular subtype was classified by applying ER, PR, and HER2 status as surrogate markers. Cases with positive ER status were classified as “luminal”, and cases with negative ER status and positive HER2 status were classified as “HER2-positive”. Cases which were negative for HER2, ER, and PR were classified as “triple negative”. Among the luminal subtypes, the cases with more than 10% of Ki-67 rate or the positive HER2 status were classified as “luminal B”, and the cases which did not satisfy both conditions were classified as “luminal A”. The cut-off point of the Ki-67 labeling index that distinguishes luminal A and B was presented at various values between 10% and 20% in previous studies [17]. In this study, the cut-off point was set at 10% for conservative evaluation.

### 2.5. Statistical Analysis

For statistical analysis, Pearson’s chi-squared test, Student’s *t* test, and the Cochran–Armitage trend test were used to evaluate any potential association between *SSBP2* expression and the clinicopathological parameters in categorical variables. Overall survival (OS) was defined as the duration from surgical treatment to death, and recurrence-free survival (RFS) was defined as the duration from surgical treatment to the first recurrence, either clinically or pathologically. The Kaplan–Meier method with a log-rank test was used to construct survival curves, and univariate and multivariate Cox proportional hazard ratio models were used to determine the significant prognostic variables. *p* values < 0.05 were regarded as statistically significant. Statistical analysis was performed using R version 3.6.2 (R Foundation for Statistical Computing, Vienna, Austria).

## 3. Results

### 3.1. Patterns of *SSBP2* Expression

**Figure 1:**
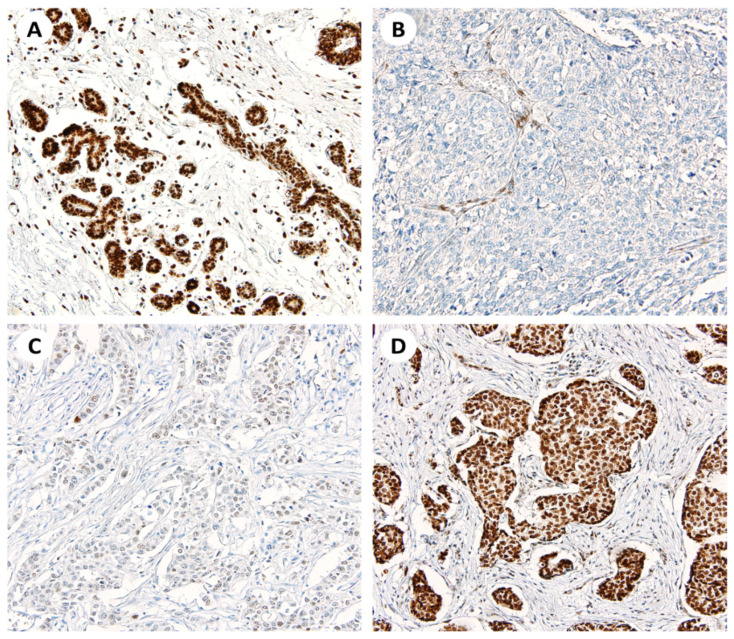
Representative photomicrographs of *SSBP2* IHC stain. (**A**) Positive nuclear stain on adjacent normal breast ductal epithelial cells (×200). (**B**) Negative stain on the tumor cells (×200). (**C**) Weak nuclear positivity on the tumor cells (×200). (**D**) Strong nuclear positivity on the tumor cells (×200).

*SSBP2* expression was evaluated on TMA slides by two pathologists. Adjacent normal breast ductal epithelial cells showed intact nuclear *SSBP2* expression. Of 491 invasive breast carcinoma cases, 61 cases (12.4%) showed negative nuclear *SSBP2* expression and 430 cases (87.6%) showed positive nuclear *SSBP2* expression on IHC staining. Representative microscopic photographs are shown in Figure 1.

### 3.2. Correlations between Nuclear *SSBP2* Expression and Clinicopathological Parameters

The correlations between nuclear *SSBP2* expression and clinicopathological parameters are summarized in Table 2. Negative nuclear *SSBP2* expression was significantly correlated with larger tumor size (*p* < 0.001, chi-squared test), higher histological grade (*p* = 0.016, Cochran–Armitage trend test), higher pT stage (*p* < 0.001, Cochran–Armitage trend test), ER status (*p* < 0.001, chi-squared test), and molecular subtype (*p* < 0.001, chi-squared test). There was no statistically significant correlation between nuclear *SSBP2* expression and age, pN stage, AJCC stage, lymph node metastasis, distant metastasis, PR status, or HER2 status. Cases of the pT1 stage were analyzed by sub-stratification into T1a, T1b, and T1c. In the *SSBP2*-positive group, 213 pT1 cases were distributed in 8 (3.8%), 30 (14.1%), and 175 (82.2%) cases in T1a, T1b, and T1c, respectively. In the *SSBP2*-negative group, 13 cases of pT1 were distributed as 0 (0%), 2 (15.4%), and 11 (84.6%), respectively. There was no significant trend in T1a, b, or c stages according to *SSBP2* expression (*p* value = 0.896, Cochran–Armitage trend test).

### 3.3. Correlations between Nuclear *SSBP2* Expression and Patient Outcomes

**Figure 2:**
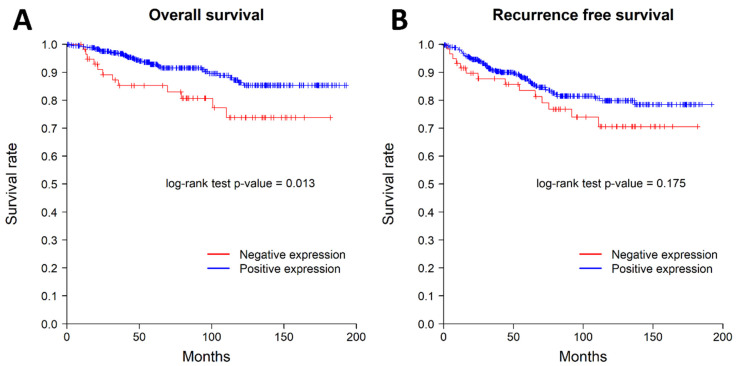
Kaplan–Meier curves for overall survival (**A**) and recurrence-free survival (**B**). The patients with loss of nuclear *SSBP2* expression showed worse overall survival; however, recurrence-free survival was not associated with nuclear *SSBP2* expression.

**Figure 3:**
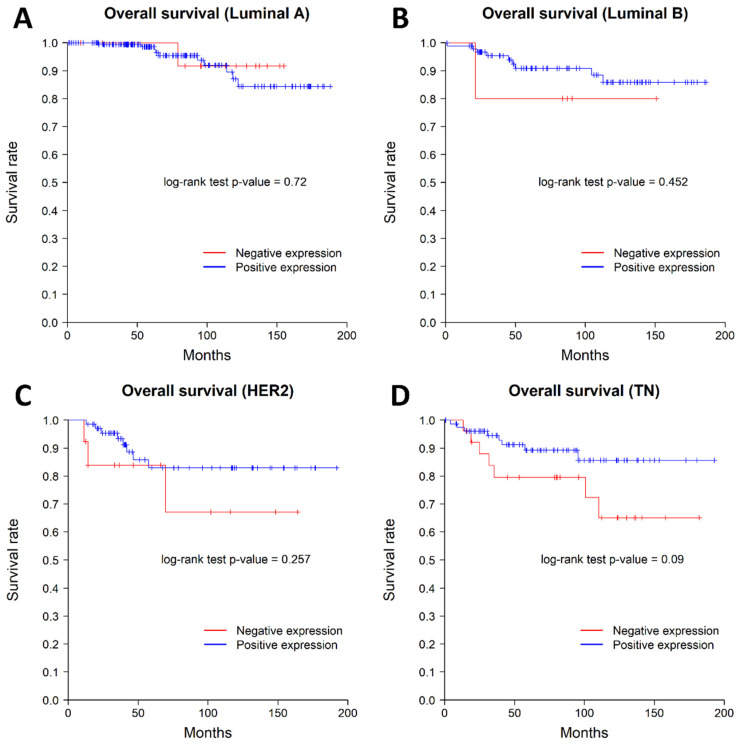
Kaplan–Meier curves for overall survival according to the molecular subtypes. (**A**) Luminal A type, (**B**) Luminal B type, (**C**) HER2 positive type, and (**D**) triple negative type. There was no difference between the *SSBP2*-positive and -negative groups in each molecular subtype of breast carcinoma.

**Figure 4:**
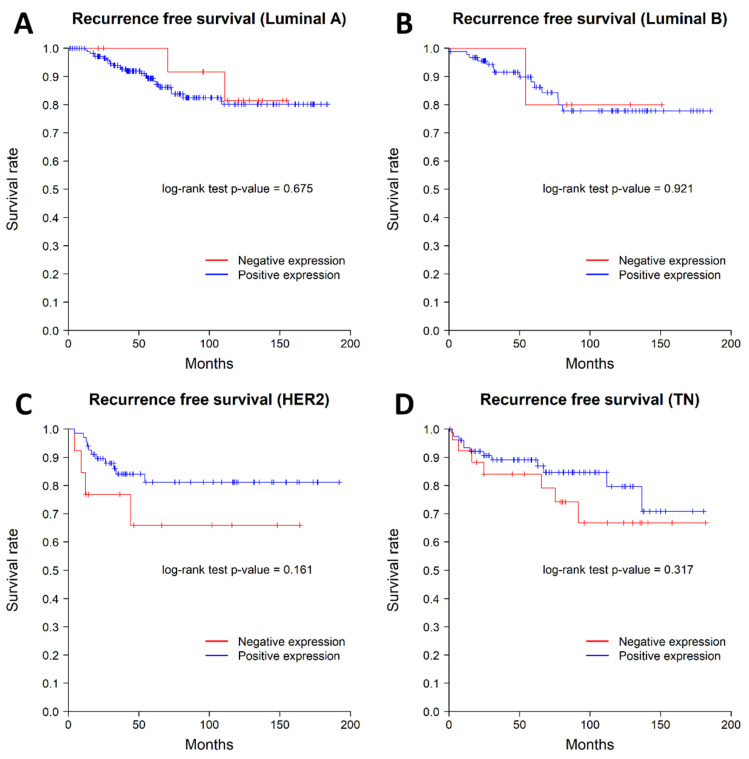
Kaplan–Meier curves for recurrence-free survival according to the molecular subtype. (**A**) Luminal A type, (**B**) Luminal B type, (**C**) HER2 positive type, and (**D**) triple negative type. There was no difference between the *SSBP2*-positive and -negative groups in each molecular subtype of breast carcinoma.

We examined the impact of nuclear *SSBP2* expression on patient survival. Nine patients with AJCC stage IV were excluded from survival analysis. Patients with negative nuclear *SSBP2* expression showed poor prognosis in OS (*p* = 0.013, log-rank test). The patients with negative nuclear *SSBP2* expression showed a tendency to have ㅁ poor RFS, but there was no statistically significant difference (*p* = 0.175, log-rank test). The Kaplan–Meier curves for OS and RFS are shown in Figure 2. The univariate Cox regression analysis for OS showed that histological grade (*p* = 0.002), pT stage (*p* = 0.01), ER status (*p* = 0.009), lymph node metastasis (*p* = 0.002), and *SSBP2* expression (*p* = 0.016) were significantly associated with OS. The multivariate Cox regression analysis showed that lymph node metastasis (*p* = 0.011) was the only independent prognostic factor for OS (Table 3). The subgroup analysis performed according to the molecular subtypes revealed no significant differences in OS or RFS. The Kaplan–Meier curves according to the molecular subtypes are shown in Figure 3 and Figure 4.

## 4. Discussion

In the present study, we evaluated nuclear *SSBP2* expression in 491 cases of invasive breast carcinoma and investigated the correlations between nuclear *SSBP2* expression and clinicopathological characteristics and patient survival. Loss of nuclear *SSBP2* expression was found in 61 cases of invasive breast carcinoma. Loss of nuclear *SSBP2* expression was significantly correlated with larger tumor size, higher histological grade, higher pT stage, ER status, and molecular subtype. In addition, loss of nuclear *SSBP2* expression was associated with poorer OS in patients with invasive breast carcinoma. In the survival analysis according to the molecular subtypes, there was no significant correlation between nuclear *SSBP2* expression and OS or RFS.

The human *SSBP2* gene was first found in leukemic blasts and is known to be deleted and translocated in acute myelogenous leukemia and myelodysplasia [15,18,19]. The *SSBP2* gene is one of three related genes with a high level of identity in deduced open-reading frames [10]. The *SSBP2* gene is a subunit of a single-stranded DNA binding complex involved in the maintenance of hematopoietic stem cells and stress response, as well as the maintenance of genome stability [7,20]. The role of *SSBP2* in cancer development and cancer progression appears to vary with the type of malignant tumor. Whether *SSBP2* is a tumor suppressor or tumor promoter is still unclear, and there is no consensus on the exact role of *SSBP2* expression in human malignancies [2]. There is also no study that supports the exact role of *SSBP2* expression in breast cancer until now. Haryono et al. reported that chromosome 5q14.1 with the *SSBP2* gene was associated with the risk of breast cancer in a pilot genome-wide association study of breast cancer susceptibility loci in Indonesia [20].

Recently, some studies have suggested that *SSBP2* shows a tumor suppressor function in human cancer. Bang et al. reported that loss of nuclear *SSBP2* expression was correlated with higher pT stage, nodal metastasis, and higher AJCC stage in gastric adenocarcinoma. They also found that loss of nuclear *SSBP2* expression was associated with poorer RFS in a microsatellite stable and Epstein–Barr virus (EBV)-negative group and HER2-negative group [14]. Chung et al. reported that loss of nuclear *SSBP2* expression was observed in 34.3% of colorectal adenocarcinoma (CRA) and 76.3% of metastatic CRA cases, and they noted that loss of nuclear *SSBP2* expression was associated with higher pT stage, vascular invasion, and poorer OS in CRA [15]. Kim et al. described loss of nuclear *SSBP2* expression in correlation with poor prognostic factors, such as larger tumor size, higher World Health Organization/International Society of Urological Pathology histological grade, tumor necrosis, sarcomatoid change, and higher pT stage, and noted it was associated with worse RFS in clear cell renal cell carcinoma [16].

Many studies have suggested that *SSBP2* is silenced through a molecular pathway mediated by promoter hypermethylation. Liu et al. reported that the *SSBP2* promoter was hypermethylated in 61.4% of prostate cancers, while benign prostatic hyperplasia had no hypermethylation in the *SSBP2* promoter [11]. Huang et al. found that *SSBP2* promoter methylation and the downregulation of *SSBP2* expression are present in 86% of esophageal squamous cell carcinoma tissue [21]. Brait et al. investigated promoter methylation frequency for 13 genes, including *SSBP2*, in ovarian cancer, and reported that 9% of ovarian cancers showed promoter hypermethylation [22]. Finally, Kagohara et al. reported that methylation in the *SSBP2* promoter was more frequently identified in adenocarcinoma than cholecystitis of the gallbladder [23].

Some authors have reported that *SSBP2* may play a role in promoting cancer progression because its expression was found to be upregulated in some tumors, including glioblastoma and hepatocellular carcinoma [12,13]. Xiao et al. reported that increased *SSBP2* expression is statistically associated with poorer OS in patients with glioblastoma [12]. Additionally, Kim et al. found that nuclear *SSBP2* expression was associated with tumor multifocality, higher histological grade, vascular invasion, and higher Ki-67 proliferation index, and they also reported that nuclear *SSBP2* expression was significantly correlated with poorer OS and RFS in patients with hepatocellular carcinoma [13].

In our study, we found that loss of nuclear *SSBP2* expression was correlated with larger tumor size, higher histological grade, and higher pT stage, which indicates that it may be involved in breast cancer progression. During survival analysis, loss of nuclear *SSBP2* expression was associated with poorer OS in patients with breast carcinoma. Our results imply that nuclear *SSBP2* expression may play a role as a tumor suppressor in breast carcinoma. However, as discussed above, previous studies reported discrepant observations regarding the association of *SSBP2* with prognosis. These discrepant reports on *SSBP2* raise the demand for further research in the context of various additional biomarkers, which are related to *SSBP2*.

There are some limitations to our study. First, we used a retrospective study design and included cases that were collected from a single center. In addition, because each case was evaluated as a single core of tissue microarray, it offered a relatively weak representation of the entire lesion. Since the proportion of cases with negative *SSBP2* expression is low in our study, routine analysis of *SSBP2* expression in every case may have weak clinical utility. However, despite this, it was confirmed that *SSBP2* had a significant effect on prognosis in this study. Therefore, further research is needed to identify some subgroups in which *SSBP2* has a more significant impact on prognosis or treatment decisions, in combination with additional biomarkers in the future.

In conclusion, we investigated the clinicopathological significance of nuclear *SSBP2* expression in 491 invasive breast carcinomas. Loss of nuclear *SSBP2* expression was significantly associated with aggressive phenotypes and poorer OS. The exact function of nuclear *SSBP2* expression and its potential as a novel biomarker for breast carcinoma should be further evaluated in future studies.

## Figures and Tables

**Table 1:** Baseline characteristics of enrolled patients (*n* = 491).

Clinicopathological Characteristics	Value (%)
Age (years, median, mean ± SD)	51, 52.9 ± 11.1
Size	
≤2 cm	226 (46.0%)
>2 cm	265 (54.0%)
Histological grade	
G1	104 (21.2%)
G2	225 (45.8%)
G3	162 (33.0%)
pT stage	
T1	226 (46.0%)
T2	223 (45.4%)
T3	28 (5.7%)
T4	14 (2.9%)
pN stage	
N0	307 (62.5%)
N1	108 (22.0%)
N2	41 (8.4%)
N3	35 (7.1%)
AJCC stage	
I	183 (37.3%)
II	207 (42.2%)
III	92 (18.7%)
IV	9 (1.8%)
LN metastasis	
Negative	307 (62.5%)
Positive	184 (37.5%)
Distant metastasis	
Negative	482 (98.2%)
Positive	9 (1.8%)
ER status	
Negative	192 (39.1%)
Positive	299 (60.9%)
PR status	
Negative	199 (40.5%)
Positive	292 (59.5%)
HER2 status	
Negative	338 (68.9%)
Positive	153 (31.1%)
Molecular subtype	
Luminal A	200 (40.7%)
Luminal B	108 (22.0%)
HER2	76 (15.5%)
TNBC	107 (21.8%)

Abbreviations: SD, standard deviation; AJCC, American Joint Committee on Cancer; LN, lymph node; ER, estrogen receptor; PR, progesterone receptor; HER2, human epidermal growth factor receptor 2; TNBC, triple-negative breast carcinoma.

**Table 2:** Correlations between *SSBP2* expression and clinicopathological parameters (*n* = 491).

Parameter		*SSBP2* Expression		*p* Value
		Positive (*n* = 430) No. (%)	Negative (*n* = 61) No. (%)	
Age (years, mean ± SD)				0.427 ^†^
		52.7 ± 10.9	54 ± 12.1	
Size				<0.001
	≤2 cm	210 (48.8%)	16 (26.2%)	
	>2 cm	220 (51.2%)	45 (73.8%)	
Histological grade				0.016 *
	G1	95 (22.1%)	9 (14.8%)	
	G2	202 (47.0%)	23 (37.7%)	
	G3	133 (30.9%)	29 (47.5%)	
pT stage				0.001 *
	T1	213 (49.6%)	13 (21.3%)	
	T2	182 (42.3%)	41 (67.2%)	
	T3	25 (5.8%)	3 (4.9%)	
	T4	10 (2.3%)	4 (6.6%)	
pN stage				0.958 *
	N0	269 (62.6%)	38 (62.3%)	
	N1	94 (21.9%)	14 (23.3%)	
	N2	37 (8.6%)	4 (6.6%)	
	N3	30 (7.0%)	5 (8.2%)	
AJCC stage				0.053 *
	I	170 (39.5%)	13 (21.3%)	
	II	172 (40.0%)	35 (57.4%)	
	III	81 (18.8%)	11 (18.0%)	
	IV	7 (1.6%)	2 (3.3%)	
LN metastasis				0.968
	Negative	269 (62.6%)	38 (62.3%)	
	Positive	161 (37.4%)	23 (37.7%)	
Distant metastasis				0.368
	Negative	423 (98.4%)	59 (96.7%)	
	Positive	7 (1.6%)	2 (3.3%)	
ER status				<0.001
	Negative	152 (35.3%)	39 (63.9%)	
	Positive	278 (64.7%)	22 (36.1%)	
PR status				0.233
	Negative	170 (39.5%)	29 (47.5%)	
	Positive	260 (60.5%)	32 (52.5%)	
HER2 status				0.374
	Negative	293 (68.1%)	45 (73.8%)	
	Positive	137 (31.9%)	16 (26.2%)	
Molecular subtype				<0.001
	Luminal A	183 (42.6%)	17 (27.9%)	
	Luminal B	95 (22.1%)	5 (8.2%)	
	HER2	71 (16.5%)	13 (21.3%)	
	TNBC	81 (18.8%)	26 (42.6%)	

Abbreviations: SD, standard deviation; *SSBP2*, single-stranded DNA-binding protein 2; AJCC, American Joint Committee on Cancer; LN, lymph node; ER, estrogen receptor; PR, progesterone receptor; HER2, human epidermal growth factor receptor 2; TNBC, triple-negative breast carcinoma. **^†^** Student’s *t* test. * Cochran–Armitage trend test.

**Table 3:** Univariate and multivariate Cox regression analyses for overall survival in patients with breast carcinoma (*n* = 482).

	Univariate Analysis			Multivariate Analysis		
	HR	95% CI	*p* Value	HR	95% CI	*p* Value
Age (per 1 year)	0.994	0.967–1.021	0.648	0.995	0.968–1.023	0.723
Histological grade 3 (vs. 1, 2)	2.48	1.398–4.4	0.002	1.578	0.832–2.994	0.163
pT stage 3, 4 (vs. 1, 2)	2.701	1.262–5.783	0.01	1.853	0.819–4.192	0.139
ER status positive (vs. negative)	0.466	0.262–0.829	0.009	0.627	0.324–1.213	0.166
HER2 status positive (vs. negative)	1.519	0.847–2.723	0.161	1.35	0.742–2.457	0.326
LN metastasis positive (vs. negative)	2.483	1.378–4.474	0.002	2.228	1.205–4.12	0.011
*SSBP2* status negative (vs. positive)	2.242	1.162–4.325	0.016	1.942	0.97–3.89	0.061

Abbreviations: HR, hazard ratio; CI, confidence interval; vs., versus; *SSBP2*, single-stranded DNA-binding protein 2; LN, lymph node; ER, estrogen receptor; HER2, human epidermal growth factor receptor 2.
